# NF-κB is weakly activated in the NOD mouse model of type 1 diabetes

**DOI:** 10.1038/s41598-018-22738-3

**Published:** 2018-03-09

**Authors:** Allison E. Irvin, Gaurang Jhala, Yuxing Zhao, Timothy S. Blackwell, Balasubramanian Krishnamurthy, Helen E. Thomas, Thomas W. H. Kay

**Affiliations:** 1 Immunology and Diabetes Unit, St Vincent’s Institute, Fitzroy, Victoria Australia; 2 The University of Melbourne, Department of Medicine, St. Vincent’s Hospital, Fitzroy, Victoria Australia; 3 Departments of Medicine and Cell and Developmental Biology, Vanderbilt University School of Medicine, Nashville, TN 37232 USA

## Abstract

Type 1 diabetes is an autoimmune disease characterised by selective destruction of pancreatic beta cells by the immune system. The transcription factor nuclear factor-kappa B (NF-κB) regulates innate and adaptive immune responses. Using gene targeting and *in vitro* analysis of pancreatic islets and immune cells, NF-κB activation has been implicated in type 1 diabetes development. Here we use a non-obese diabetic (NOD) mouse model that expresses a luciferase reporter of transcriptionally active NF-κB to determine its activation *in vivo* during development of diabetes. Increased luciferase activity was readily detected upon treatment with Toll-like receptor ligands *in vitro* and *in vivo*, indicating activation of NF-κB. However, activated NF-κB was detectable at low levels above background in unmanipulated NOD mice, but did not vary with age, despite the progression of inflammatory infiltration in islets over time. NF-κB was highly activated in an accelerated model of type 1 diabetes that requires CD4^+^ T cells and inflammatory macrophages. These data shed light on the nature of the inflammatory response in the development of type 1 diabetes.

## Introduction

Type 1 diabetes is caused by a breakdown of immune tolerance resulting in infiltration of the pancreatic islets by immune cells and the subsequent destruction of insulin-producing beta cells. This is a complex process involving coordination between many cell types. How this process is regulated is not thoroughly understood. The interaction between immune cells and beta cells is mediated by proinflammatory cytokines such as IFN-γ, and other immune signals, including TCR/MHC complexes and co-stimulation^1,2^.

NF-κB is a family of transcription factors involved in a diverse range of physiological processes, but is particularly important in regulation of the immune system. It is essential for normal development and maintenance of the immune system, as well as activation, proliferation and differentiation of both innate and adaptive immune cells in response to infection. NF-κB is activated in response to many of the immune signals present in the inflamed islet^3^.

Dysregulated NF-κB has been implicated in inflammatory diseases including asthma^4^, rheumatoid arthritis^5^ and inflammatory bowel disease^6^. In type 1 diabetes, an overactive NF-κB response was observed in antigen-presenting cells (APCs) of non-obese diabetic (NOD) mice in response to Toll-like receptor (TLR) ligands^7–9^. Deficiency of NF-κB c-Rel resulted in accelerated diabetes in NOD mice, as a result of impaired regulatory T cell function in the absence of c-Rel^10^. In beta cells, the participation of NF-κB in disease varies depending on the diabetes model. Diabetes was accelerated in NOD mice with a super-repressed NF-κB expressed specifically in beta cells^11^, suggesting NF-κB plays an anti-apoptotic role. In contrast, conditional blockade of NF-κB in beta cells prevented diabetes in the multiple low-dose streptozotocin dependent (MLDS) model of diabetes suggesting a pro-apoptotic role^12^. Despite this evidence for a role of NF-κB in the development of diabetes, the extent of activated NF-κB in the natural progression of diabetes in NOD mice has not yet been characterised *in vivo*.

We developed a transgenic NOD mouse model (NOD.NGL) that contains a luciferase reporter of activated NF-κB. All active NF-κB dimers can bind to the specific κB responsive element^13^, meaning that expression of the reporter gene is a reflection of the total transcriptional ability of NF-κB. This reporter has been used successfully in mice of other genetic backgrounds to study *in vivo* activation of NF-κB in inflammatory disease^14,15^. Our results show that while NF-κB transcriptional activity was observed in the islets of NOD mice, this was at low levels compared to that observed in a more acute, accelerated CD4^+^ T cell-dependent model of diabetes.

## Results

### Activated NF-κB is detectable after *in vitro* stimulation of macrophages and DCs

**Figure 1:**
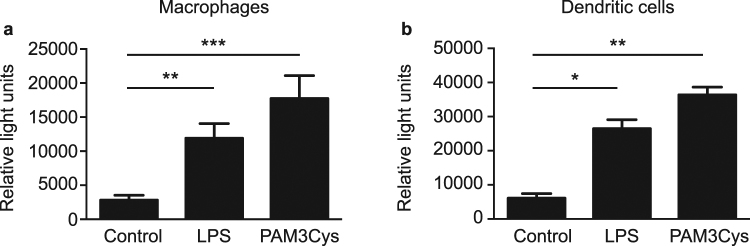
Luciferase expression is activated by TLR ligands in NOD.NGL antigen presenting cells. (**a,b**) Luciferase assay of bone marrow-derived macrophages (**a**) or DCs (**b**) cultured with 10 ng/mL LPS or 10 ng/mL PAM_3_Cys for four hours. Results are displayed as mean ± SEM of n = 4 and n = 3 independent experiments for A and B respectively. *p < 0.05, **p < 0.01, ***p < 0.001, one-way ANOVA.

We first confirmed that the NGL transgene and luciferase reporter were functional. Bone marrow-derived macrophages and DC from NOD.NGL mice were cultured with the TLR ligands LPS or PAM_3_Cys for four hours to activate NF-κB, after which a luciferase assay was performed. In DC and macrophage populations there was a significant increase in luciferase expression after treatment with either TLR ligand in comparison to control cells, indicating NF-κB activation (Fig. 1). We were unable to detect GFP expression either by flow cytometry or fluorescent microscopy (data not shown), suggesting that, unlike on a C57BL6/DBA background^14^, this component of the transgene was repressed in our NOD.NGL mice.

### NF-κB is activated *in vivo* by LPS stimulation

**Figure 2:**
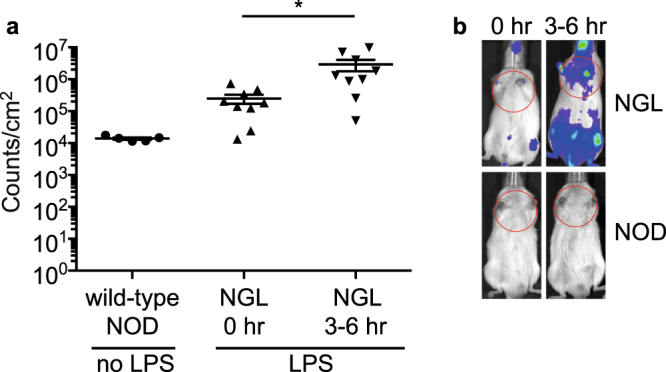
Luciferase expression after *in vivo* injection of LPS. (**a**) NGL mice (n = 9) were injected with LPS and imaged in the IVIS immediately (0 hr) or 3–6 hours post injection. Untreated wild-type NOD mice (n = 5) were analysed as controls. Data show individual mice with mean ± SEM. *p = 0.01, one-way ANOVA. (**b**) Representative IVIS images for wild-type NOD and NGL mice at 0 and 3–6 hours post LPS injection, showing regions analysed in red circles.

We next tested whether NF-κB activation could be detected *in vivo*. Using a well-characterised model of NF-κB activation, we injected LPS into mice and imaged them for bioluminescence after 3–6 hours. LPS stimulated significant bioluminescence in all mice (Fig. 2), indicating that the luciferase transgene is functional *in vivo*.

### NOD.NGL mice develop spontaneous diabetes

**Figure 3:**
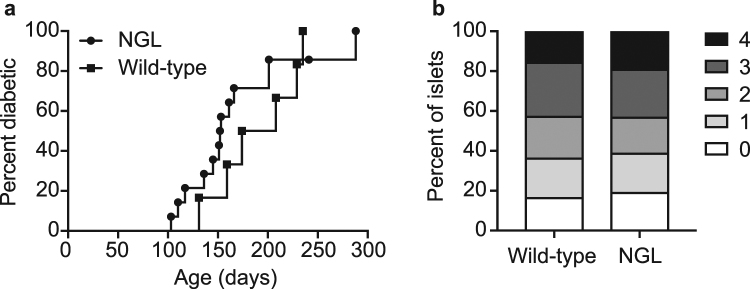
Diabetes incidence in NOD.NGL mice. (**a**) Diabetes was monitored in female NGL (n = 14) and wild-type NOD littermates (n = 6) until 300 days of age. Data are not significantly different, log-rank test. (**b**) Insulitis scores of NGL (n = 12) and wild-type NOD littermates (n = 5) of mice 100–150 days of age. Data are not significantly different, two-way ANOVA.

The incidence of spontaneous diabetes was examined in NOD.NGL mice. Mice developed diabetes at the same incidence and rate as wild-type non-transgenic littermates (Fig. 3a) and pancreatic infiltration (insulitis) was no different from NOD mice (Fig. 3b). These results confirm that the transgene did not affect disease development, making this a valid model for studying NF-κB activation in spontaneous disease.

### NF-κB is weakly activated during spontaneous diabetes development in NOD mice

**Figure 4:**
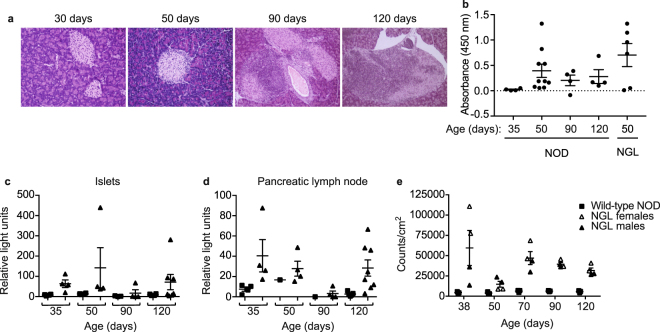
NF-κB activation in islets and PLN during development of spontaneous diabetes in NOD mice. (**a**) Representative H&E staining of islets from female NOD mice at the ages indicated. Magnification 200×. (**b**) Insulin autoantibodies (IAA) in the serum of female NOD or NGL mice at the ages indicated. Data not significantly different (one-way ANOVA). (**c,d**) Islets or PLN were isolated from female NOD and NOD.NGL mice at 35, 50, 90 and 120 days of age, corresponding to checkpoints in insulitis development. Luciferase assays were performed on (**c**) 200 islets or (**d**) 2–3 million PLN cells from NOD mice (n = 1–5), or NGL mice (n = 4–8). Data show individual mice with mean ± SEM. Differences in luminescence between NOD and NOD.NGL samples were statistically significant with p = 0.03 in islets and p = 0.04 in PLN. There was no statistically significant impact of age on luminescence in either the islets or PLN (two-way ANOVA). (**e**) Luminescence detected using the IVIS from the pancreas region. Luminescence was significantly greater in the male and female NGL mice (n = 4) compared to the NOD mice (n = 4) (p < 0.001) but there was no statistically significant effect of age (p = 0.65, two-way repeated measures ANOVA).

To examine NF-κB activation during the development of insulitis and progression to autoimmune diabetes, islets and pancreatic lymph nodes (PLN) were isolated from NOD.NGL mice and a luciferase assay was performed immediately *ex vivo*. NF-κB activation was examined at key checkpoints in the development of diabetes: before the onset of insulitis (35 days), after the onset of insulitis (50 days), during progression of lymphocyte infiltration (90 days) and in the pre-diabetic stage (>120 days). Although the timing of development of hyperglycaemia is variable in NOD mice, and more common in females, pancreatic inflammation and autoimmunity increases gradually over this time course. Representative histological sections show the increasing islet inflammation at the different time points in female NOD mice (Fig. 4a). Insulin autoantibodies (IAA) were not detected at 35 days of age but were present at the other time points, including in 50 day old NGL mice (Fig. 4b). While luciferase activity in NOD.NGL tissues was significantly higher than background levels seen in NOD tissues (islets p = 0.03, PLN p = 0.04), luciferase expression was low (<500 RLU) and was variable between mice at each age tested (Fig. 4a,b). Overall, age had no significant impact on luciferase expression (islets p = 0.47, PLN p = 0.31, two-way ANOVA), indicating that the variability in disease progression did not significantly affect NF-κB activation.

To visualise activated NF-κB in whole mice during diabetes development, bioluminescence imaging was performed using the IVIS. Bioluminescence was examined at 35, 70, 90 and 120 days in the same two female and two male NOD.NGL mice, and in three NGL-negative littermate controls (Fig. 4c). Consistent with the luciferase assays from isolated islets and PLN, low levels of luciferase were detectable in the pancreas area at any age (<1.5 × 10^5^ counts/cm^2^). A significant difference was observed in the luminescence detected from NOD.NGL mice compared to background luminescence in the NOD controls (p < 0.001, two-way repeated measures ANOVA). No effect was seen with age (p = 0.65).

### NF-κB is highly activated in an accelerated model of type 1 diabetes

**Figure 5:**
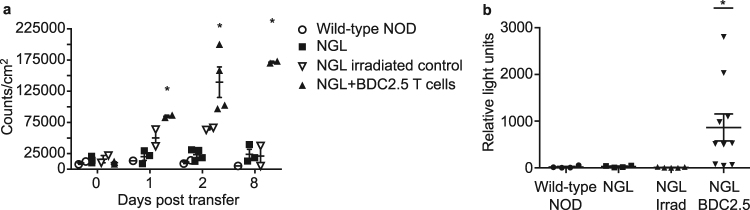
NF-κB is activated after adoptive transfer of diabetogenic BDC2.5 T cells. (**a**) NOD.NGL mice (n = 2–4) were irradiated (900 R) then adoptively transferred 1 × 10^6^ purified diabetogenic BDC2.5 T cells. IVIS imaging was performed on the day of transfer (day 0), and 1, 2, and 8 days post-transfer. Wild-type NOD mice (n = 1–2), unirradiated NGL mice (n = 3) and irradiated NGL mice that did not receive BDC2.5 T cells (n = 2) were included as controls. Data show mean ± SEM. *p < 0.01, one-way ANOVA. (**b**) Eight days after transfer 200 islets from NOD mice (n = 4), NGL mice (n = 4), irradiated NGL controls (n = 5) or irradiated NGL that also received BDC2.5 T cells (n = 10) were assayed for luciferase activity *p < 0.001, log-transformed data, one-way ANOVA.

The low levels of activated NF-κB detected in islets of NOD.NGL mice may be because development of insulitis and diabetes in NOD mice is slow and not synchronous. To determine whether NF-κB is activated in an accelerated and synchronous model of diabetes, diabetogenic BDC2.5 T cells were adoptively transferred into irradiated NOD.NGL mice. In this model, all mice develop diabetes within 11 days^16^. Luminescence appeared in the abdominal region as early as one day after irradiation. This was most likely radiation-induced NF-κB activation as luminescence was also detected in irradiated mice that received no T cells. By day 8, luminescence had decreased in the irradiated controls, but in the mice that received BDC2.5 T cells, luminescence increased and also localised to the pancreas region (Fig. 5a). Luciferase assays of whole islets isolated at day 8 showed that NF-κB activation in islets was significantly higher in mice that received BDC2.5 T cells than in the unmanipulated or irradiated control mice (Fig. 5b) (p < 0.001, one-way ANOVA, log transformed data).

## Discussion

The methods available for studying NF-κB activation *in vivo* have so far been limited, and thus the timing and magnitude of NF-κB activation in the islets in spontaneous autoimmune diabetes has not been formally assessed. Here we have developed a model for studying *in vivo* activation of NF-κB using a luciferase reporter in the NOD mouse model of diabetes. NF-κB is critical in controlling the activation and proliferation of immune cells. Furthermore, the infiltrated islet is often thought of as a highly inflammatory environment due to the presence of cytokines. Therefore, we expected that high levels of NF-κB activation would be present in the islets. In contrast, we found that levels of activated NF-κB were low during progression to spontaneous diabetes in NOD mice, while it was more highly activated in an accelerated and synchronous model of diabetes.

NOD mice with NF-κB signalling deficiency develop accelerated diabetes, suggesting that NF-κB plays a protective role in this model^10,11^. Our data suggest that the level of NF-κB we observed in NOD islets is sufficient for protection from the inflammatory environment that caused this accelerated beta cell destruction. Examination of the mRNA expression profile of inflamed NOD islets did not identify classical NF-κB-dependent genes, such as TNF-α, IκBα, A20 or Fas^17,18^. Instead, the destruction of beta cells in inflamed islets appears to be dominated by IFN-γ and IFN-induced genes^18–20^. These findings are consistent with the idea that NF-κB is weakly activated in beta cells in NOD mice.

NF-κB is typically associated with acute inflammation and is critical in the initiation of immune responses against infections, or in acute graft rejection. The low level of NF-κB activation observed in NOD mice is consistent with the idea that the immune response in autoimmune diabetes is not an acute one. Stringent negative regulatory pathways ensure that NF-κB is rapidly down-regulated after initial activation and it is only in selective circumstances that NF-κB is persistently activated^21^. It is expected that these negative regulatory mechanisms would keep activated NF-κB levels to a minimum in NOD islets. In addition, islet destruction in spontaneous diabetes is not a synchronous process, taking place over many months in NOD mice. If NF-κB is more highly activated at a particular stage in insulitis or beta cell destruction, the signal may be diffused by the lack of synchronicity between different individual islets in the pancreas.

In contrast to spontaneous diabetes in NOD mice, higher levels of NF-κB activation were seen after adoptive transfer of BDC2.5 T cells into irradiated NOD.NGL mice, correlating with synchronous and acute destruction of islets. The increase in NF-κB activation in this model is also consistent with gene expression studies performed using islets from BDC2.5 mice, where expression of NF-κB-regulated genes including Fas, chemokines and cytokines is increased in beta cells^20^. It remains possible that the high levels of NF-κB activation originate from either beta cells or macrophages, or both, in this model, and this remains to be determined.

Evidence from human pancreases from type 1 diabetes sufferers suggests that islet destruction also occurs over a long period of time, with very little immune infiltration compared with the NOD mouse^22^. Our data suggest that NF-κB activation may be low over this long time-frame in human type 1 diabetes. Our observation that NF-κB was activated in an accelerated model of diabetes suggests it may have a role in the acute destruction of beta cells. Inhibitors of NF-κB may therefore be a therapeutic option in preventing islet destruction in transplantation settings, but of limited use in progression of spontaneous diabetes.

## Methods

### Mice

Mice were bred and housed at the Bioresources Centre at St Vincent’s Hospital, Fitzroy. Animal experimentation was carried out with approval from the St Vincent’s Hospital animal ethics committee, in accordance with the institutional guidelines and regulations. Transgenic NOD reporter mice were generated using the NGL construct containing four tandem copies of the 5′ HIV-long terminal repeat enhancer (with two NF-κB binding sites, GGGACTTTCC) upstream of an enhanced GFP-luciferase fusion protein as previously described^14^. The purified NGL construct was injected into fertilized NOD ova and reimplanted into foster mothers using standard techniques in the transgenic mouse facility at the Walter and Eliza Hall Institute. Males containing the NGL transgene were mated with wild type NOD females. All offspring were genotyped by PCR. NOD.BDC2.5 mice contain a transgene encoding an MHC class II-restricted TCR that recognises chromogranin A^23^.

### Isolation and activation of macrophages and dendritic cells

Bone marrow-derived macrophages were obtained by culturing bone marrow cells in 10 ng/mL GM-CSF for 5–7 days. For expansion of dendritic cells (DC), 10 ng/mL of mouse IL-4 was also added to culture medium. To activate NF-κB, bone marrow macrophages or DC were incubated with 10 ng/mL LPS or 10 ng/mL PAM_3_Cys in complete RPMI for 4 h, then harvested for luciferase assays by incubating with 5 mM EDTA for 15 min at 37 °C and washing with PBS.

### Islet isolation

Islets were isolated from mice as previously described^24^. After gradient purification, islets were immediately pelleted and lysates prepared for luciferase assays.

### Luciferase assays

Luciferase assays were performed using the Luciferase Assay System (Promega). Cell lysates were prepared by adding 60 μL of reporter lysis buffer to pellets of whole islets, a single cell suspension of pancreatic lymph node cells, macrophages or DC. After one freeze-thaw cycle, samples were centrifuged at 4 °C for 5 min at 13,200 rpm. Cell lysates were collected and plated into an opaque 96 well plate (Nunc) in triplicate measures of 20 μL. To perform the luciferase assay, 100 μL of luciferase assay substrate (Promega) was added to each sample and luminescence was measured over 5 sec using a microplate luminometer with automatic injector (PolarStar). Measurements are given in relative light units (RLU).

### *In vivo* LPS stimulation

LPS was obtained from Sigma-Aldrich, and delivered i.p. at a dose of 3 µg/g body weight. Immediately after, and at 3–6 hours after LPS, mice were injected with luciferin and imaged by *in vivo* bioluminescence imaging. Quantification of luciferase activity was measured over the chest region as indicated in Fig. 2b.

### *In vivo* bioluminescence imaging

Mice were anaesthetised using 1.5% isoflurane and arranged in the Xenogen IVIS-200 (PerkinElmer, Waltham, MA, USA). D-luciferin potassium salt (Caliper LifeSciences) was injected i.p. at 150 mg/kg and luminescence was measured at time points 1–15 min post-injection. Ventro-dorsal and lateral arrangements were used. Data were analysed using LivingSystem software (Xenogen). The location of the abdominal region over the pancreas was established using MIP-Luc-VU mice expressing luciferase in beta cells under control of the mouse insulin promoter^25^ and the same region was analysed in NOD.NGL mice and photon counts per cm^2^ were calculated.

### Diabetes, insulitis and autoantibodies

Female mice were monitored for diabetes for 300 days. Mice with two consecutive blood glucose measurements of ≥15 mmol/L were considered diabetic. Insulin autoantibodies (IAA) were measured with a 96-well filtration plate micro-IAA assay as described previously^26^. For immunohistochemistry, tissues were fixed in neutral buffered formalin and embedded in paraffin. Sections (5 µm) were stained with haematoxylin and eosin or insulin for insulitis scoring as previously described^27^ using the following scale: 0 = no infiltrate, 1 = peri-islet infiltrate, 2 = extensive (>50%) peri-islet infiltrate, 3 = intra-islet infiltrate, and 4 = extensive intra-islet infiltrate (>80%) or total beta-cell loss.

### Adoptive transfer of diabetogenic BDC2.5 T cells

Spleens from NOD.BDC2.5 mice were dispersed through a 70 µm cell strainer (BD Biosciences), then cells were stained with antibodies recognising CD4, CD25 and the clonotypic BDC2.5 TCR^28^. Live BDC2.5^high^CD4^+^CD25^−^ cells were sorted on a FACS Aria (BD Bioscience, San Jose, CA, USA). NOD.NGL mice of 8–10 weeks of age were irradiated (9 Gy) and 1 × 10^6^ cells/mouse were injected into the tail vein. Bioluminescent imaging was performed as described above. Recipient mice were sacrificed at 8 days post-transfer for luciferase assays of the isolated islets.

### Statistical analysis

Data were analysed using GraphPad Prism software (GraphPad Software Inc., San Diego, CA). Repeated one- or two-way analysis of variance (ANOVA) was used to determine statistical significance. Values are given as mean ± SEM. Diabetes development was analysed by the log-rank test.

### Data availability

All data generated or analysed during this study are included in this published article.
